# Does the motor cortex differentiate between linguistic symbols and scribbles?

**DOI:** 10.3389/fnhum.2013.00715

**Published:** 2013-10-24

**Authors:** Luca F. Ticini

**Affiliations:** Wellcome Laboratory of Neurobiology, University College LondonLondon, UK

**Keywords:** EEG, mu-rhythm, sensorimotor activation, reading, symbols

How the human brain distinguishes between linguistic symbols (i.e., letters and characters) and scribbles is not entirely understood. Some experimental evidence indicates the existence of a specialized visuo-motor network (comprising the left premotor cortex) that serves the perception of written language symbols as well as their production (e.g., Anderson et al., 1990; Starrfelt, 2007). Damage to this network impairs the identification of single symbols whilst sparing the ability to read, for instance, numbers.

In a recent paper, Heimann et al. (2013) used Electroencephalography (EEG) to deepen our understanding of the role of visuo-motor cortices in handwriting categorization. In their experiment, Heimann et al. asked individuals to observe Roman letters (belonging to the alphabet of the participant's mother language), Chinese (unfamiliar) characters, and scribbles (judged by the participants as not being linguistic symbols). As a marker of sensorimotor activity, they used the central alpha event-related desynchronization in the EEG (ERD; see Pfurtscheller and Lopes da Silva, 1999), which describes the contralateral attenuation of the EEG power during preparation and execution of movements (Jasper and Penfield, 1949; Chatrian et al., 1959; Pfurtscheller and Berghold, 1989).

On the one hand, Heimann and colleagues found that all handwriting categories evoked activity in the observer's cortical motor system (see their Figure 3). This first result suggests that the brain interpreted the handwritings “motorically” as traces of hand movements executed by another individual (see also Umiltà et al., 2012), regardless of whether they were language symbols (i.e., Roman letters and Chinese characters) or scribbles, and regardless of whether the observer was familiar or trained in the handwriting style. On the other hand, the time-course of the ERD (see their Figure 6) showed that scribbles evoked less sensorimotor activity than linguistic symbols. More specifically, alpha desynchronization (ERD) was generally weaker and resynchronization (return to the baseline level) faster for scribbles than for symbols. Why was this so? Heimann et al. hypothesized that this “might depend on either sensory-motor training in a broad sense, separating symbols in general from scribbles, or on visual features allowing a categorical distinction between symbols and scribbles. … Since a further major difference among the employed stimuli is their symbolic value, present in Roman letters and Chinese characters and absent in scribbles, central alpha ERD modulation in the temporal domain could depend on visual features marking the stimuli as linguistic symbols” (my ellipsis; see also Longcamp et al., 2005 and Wong et al., 2008). As far as the first consideration (sensorimotor training) is concerned, it is improbable that the difference observed between scribbles and symbols can be explained by previous experience, since the participants were indeed experienced in Roman letters but naïve to Chinese characters (see the results of their Rating task). The second consideration supports a crucial role of visuo-motor associations in the ability to read language symbols (see also Freyd, 1983; Babcock and Freyd, 1988; Longcamp et al., 2003, 2006, 2008; James and Gauthier, 2009) and suggests the presence of visual features in symbols that “are the outcome of the way they are written and which precisely make them recognizable as symbols (not scribbles).” In the present article, I argue that a difference between the visual features of the two groups of writings does exist, but that it is not necessarily language-related. Indeed, although the three groups of handwritings were matched in size and stroke-thickness, mere visual inspection reveals that the scribbles contain less ink (or number of pixels, NP; see their Figure 1S). The stimuli being matched, the difference in NP necessarily signifies that the length or the number of strokes differed between the stimuli. This of course may have affected the results by providing the participants with dissimilar information about the hand actions that had been executed to draw the handwritings.

**Figure 1:**
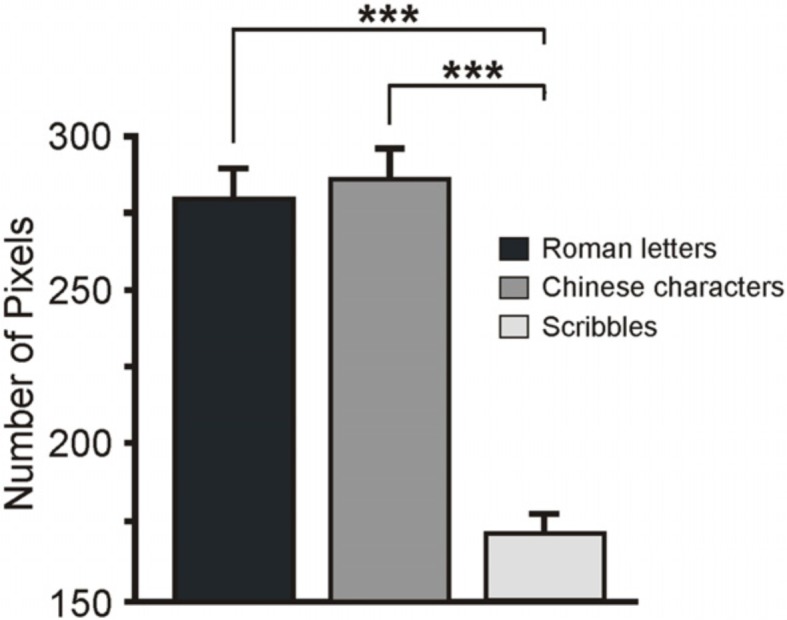
**Mean number of pixels in the three categories of stimuli**. The scribbles had a number of pixels significantly inferior to symbols. Error bars represent S.E.M. ^***^p < 0.001.

To quantify and compare the amount of ink in the 3 categories of handwritings (20 Roman letters, 20 Chinese characters and 20 scribbles), I analyzed the differences in their NP. First, I identified the contours of each handwriting with Color Photo Paint (Corel Draw 9) using the option Color Mask (tolerance = 30, smoothing = 0, black color). Next, I calculated the total NP with the option Histogram. A One-way ANOVA with the factor Category (letters, characters, scribbles) showed a main effect, *F*_(2, 57)_ = 52.49, *p* < 0.001, η_p_^2^ = 0.65, indicating that the NP differed between the types of handwritings (a significance threshold of *p* < 0.05 was set for all statistical tests). *Post-hoc* comparisons (Newman-Keuls's *post-hoc* test) revealed that NP between Roman letters (280.1 ± 9.5; Mean ± S.E.M.) and Chinese characters (286.2 ± 9.9) did not differ (*p* = 0.63; see Figure 1). Instead, their NP was significant larger (*ps* < 0.001) than the NP in the scribbles (172.2 ± 6.8). After exclusion of the Roman letter “J”—that according to Heimann et al. “was not definitely recognized by more than 50% of participants” as a symbol of a written language, and that was consequently excluded from their EEG analysis—the results remained unchanged: the ANOVA showed a main effect of Category, *F*_(2, 54)_ = 51.31, *p* < 0.001, η_p_^2^ = 0.65, while *post-hoc* tests indicated the equivalence of Roman letters and Chinese characters (*p* = 0.42) and their difference from the scribbles (*ps* < 0.001).

The result of this analysis demonstrates that the three handwriting categories in Heimann et al. differed for low-level visual features: i.e., the NP was larger for letters and characters than for scribbles. Interestingly, this difference did not have an impact on the activity of visual areas, perhaps because the stimuli were matched in size and stroke-thickness. Nonetheless, it may have affected motor activity. Indeed, the difference in the length/number of strokes may affect the pattern of results by, for instance, providing the participants with less information about the hand gestures that produced the scribbles (and consequently eliciting a weaker and short lasting ERD). I therefore suggest that symbols and scribbles can be differentiated on the basis of visual features that are not necessarily attributable to the differences between language and non-language writings, as argued by the authors.

Interestingly, Heimann et al. found strong differences in ERD in the right hemisphere between Roman letters and scribbles but not between Chinese characters and scribbles (especially in the later epochs). Otherwise, in the left (dominant) hemisphere, symbols and scribbles differed in earlier epochs whilst in later epochs the larger difference was found between characters and scribbles, and not between letters and scribbles. Although difficult to interpret, this pattern suggests that Chinese characters cannot be fully considered equivalent to Roman letters and that multiple factors besides language coding and NP could explain the different modulation of motor activity for the three categories of handwritings. Further research in this direction is needed.

In conclusion, the timely paper by Heimann and colleagues furthers our understanding of the action-perception coupling and motor involvement that takes place during the perception of handwritings. However, the difference spotted in low-level visual features between symbols and scribbles wakens the hypothesis that the motor cortex would be able to recognize Roman letters and Chinese characters based on their appearance as linguistic symbols.
